# Protective effects of Apelin-13 on nicotine-induced H9c2 cardiomyocyte apoptosis and oxidative stress

**DOI:** 10.18332/tid/201400

**Published:** 2025-03-18

**Authors:** Can Xu, Xinyu Nie, Ru Xu, Luyang Zhou, Dongjin Wang

**Affiliations:** 1 Department of Cardiac Surgery, Nanjing Drum Tower Hospital, Nanjing University Medical School, Nanjing, People’s Republic of China; 2 Nanjing University Medical School, Nanjing, People’s Republic of China; 3 Department of Anesthesiology, Nanjing Drum Tower Hospital, Nanjing University Medical School, Nanjing, People’s Republic of China

**Keywords:** apelin-13, apoptosis, cardiomyocyte, nicotine, oxidative stress

## Abstract

**INTRODUCTION:**

We aimed to explore the role of Apelin-13 in resisting oxidation, inflammation as well as apoptosis and its underlying mechanisms of action using a model of nicotine-induced H9c2 cardiomyocyte injury.

**METHODS:**

H9c2 cardiomyocytes were randomly divided into control, nicotine, nicotine + Apelin-13, and Apelin-13 groups. Cell counting kit-8 assay was conducted to determine the cell viability. Interleukin (IL)-6, superoxide dismutase, tumor necrosis factor-alpha (TNF-α), glutathione peroxidase (GSH-Px), IL-β, catalase (CAT), IL-8, lactate dehydrogenase (LDH), and malondialdehyde (MDA) levels were examined. A 2',7'-dichlorodihydrofluorescein diacetate assay was conducted to measure the intracellular reactive oxygen species (ROS) level. The morphology of apoptotic cardiomyocytes was observed by 4',6-diamidino-2-phenylindole staining. Western blotting was employed to measure the protein expressions of apoptotic factors B-cell lymphoma-2 (Bcl-2) and Bcl-2-associated X (Bax). Apoptosis was quantified using Annexin V/propidium iodide staining.

**RESULTS:**

Exposure of H9c2 cardiomyocytes to 10 μM nicotine significantly reduced cell viability and increased LDH release, oxidative stress (elevated MDA and ROS levels with decreased superoxide dismutase, GSH-Px, and CAT activities), pro-inflammatory cytokines (IL-6, TNF-α, IL-1β, IL-8), and apoptotic markers (increased Bax with decreased Bcl-2 expression, along with nuclear condensation) (p<0.05). In contrast, treatment with 2 μM Apelin-13 significantly alleviated these deleterious effects, enhancing cell viability, restoring antioxidant enzyme activities, reducing oxidative and inflammatory responses, and inhibiting apoptosis (p<0.05).

**CONCLUSIONS:**

Nicotine induction increases the oxidative stress and apoptotic capacity of H9c2 cardiomyocytes, but Apelin-13 protects H9c2 cardiomyocytes against nicotine-induced apoptosis and oxidative stress.

## INTRODUCTION

Cardiovascular diseases are one of the leading causes of sudden human death^1,2^. They have high incidence and mortality rates^3^. Myocardial infarction atherosclerosis, heart failure, hypertension, and many other cardiovascular diseases have been related with oxidative stress^4^. An excess of reactive oxygen species (ROS) is generated under oxidative stress, which is a vital link during cardiovascular disease progression^5^. ROS produced in excess not only severely damage cardiomyocytes and disrupt the balance between oxidative and anti-oxidant systems^6^, but also trigger the oxidative modification of cellular macromolecules, thereby suppressing protein function and facilitating apoptosis^7^. Cardiomyocytes are non-renewable, and some of them die of oxidative stress and apoptosis, eventually resulting in the deterioration of cardiac function or even death.

Nicotine, a major component of tobacco, has an association with the increased risk of cardiovascular diseases^8,9^. Nicotine promotes hypertension-related vascular endothelial dysfunction, oxidative stress, inflammation, fibrosis and apoptosis, and has influences on the structure and function of the heart^10^. Apelin, a bioactive peptide isolated from bovine stomach extract, works as the endogenous ligand of angiotensin-receptor type 1, a human G protein-coupled receptor^11^. Apelin is essential for the development of blood vessels as well as the heart, and its existence in perivascular areas during early embryogenesis has been verified. Among various subtypes of Apelin, Apelin-13 is the most abundant and potent in the heart^12^. It participates in cardiac development, protects against ischemia-reperfusion injuries, cardiac fibrosis and remodeling and regulates blood pressure to trigger vasodilation, and its mechanical function enhances the contractility of healthy and failing hearts^13^.

In this study, a model of nicotine-induced cardiomyocyte injury was used to investigate the role of Apelin-13 in resisting oxidation, inflammation as well as apoptosis and its underlying mechanism of action.

## METHODS

### Culture and treatment of cells

The American Type Culture Collection supplied rat embryo-derived H9c2 cardiomyocytes, which were preserved under a humidified condition (5% CO_2_ and 95% air) at 37°C using Dulbecco’s modified Eagle medium supplemented with penicillin/streptomycin (1%) plus fetal bovine serum (10%). Two days prior to experiments, they were inoculated on glass coverslips to achieve a confluence of ≥90%.

H9c2 cardiomyocytes were evenly divided into a control group, a nicotine group, a nicotine + Apelin-13 group, and an Apelin-13 group. The control group was not subjected to any treatment. The nicotine group underwent 48 h of nicotine treatment at the concentrations of 0.1, 10 and 100 μM, with10 μM nicotine finally selected for subsequent experiments. The nicotine + Apelin-13 group was co-cultured with 10 μM nicotine and Apelin-13 (0, 0.1, 1, 2 and 4 μM) for 48 h. Based on cell viability, 2 μM Apelin-13 was selected for subsequent experiments. The Apelin-13 group was cultured with 2 μM Apelin-13 for 48 h.

### Cell counting kit-8 (CCK-8) assay

The viability of cells in different groups was examined by CCK-8 assay. In brief, a 96-well plate was utilized for 48-h inoculation of H9c2 cardiomyocytes (2×10^4^/well in density). Afterwards, complete Dulbecco’s modified Eagle medium was employed to substitute the culture supernatant, and the cells were added CCK-8 stock solution at 10 μL/well, followed by 2 h of 37°C incubation. Then, all wells were supplemented with DMSO in a volume of 150 μL after removing the medium. Lastly, Multiskan GO microplate spectrophotometer (Thermo Fisher Scientific, USA) was employed to measure the optical density at 490 nm.

### Biochemical analysis

After plate (24 wells) inoculation of H9c2 cardiomyocytes (1×10^5^/mL in density), the culture supernatant in each well was obtained in a volume of 100 μL, and colorimetric assay was conducted to detect the lactate dehydrogenase (LDH) activity using an assay kit. Then cold phosphate-buffered saline (PBS) washing together with 10 min of 300g centrifugation was performed for H9c2 cardiomyocytes. Subsequently, the precipitate underwent ultrasound pulverization with the supernatant discarded. Next, the cell lysate was resuspended. Afterwards, the levels of interleukin-6 (IL-6), superoxide dismutase (SOD), tumor necrosis factor-alpha (TNF-α), glutathione peroxidase (GSH-Px), IL-β, malondialdehyde (MDA), IL-8, and catalase (CAT) were detected by the microplate reader according to the protocols of corresponding kits. In addition, colorimetric assay and xanthine oxidase assay were carried out to determine GSH-Px activity and SOD activity, respectively, and thiobarbituric acid assay was performed to measure MDA level. Moreover, CAT activity was detected by optical spectrophotometry, and IL-6, TNF-α, IL-8 as well as IL-β levels were examined through enzyme-linked immunosorbent assay.

### Measurement of ROS level in cells

A 2’,7’-dichlorodihydrofluorescein diacetate (DCFH-DA) assay was carried out to determine the ROS level in cells. To be specific, DCFH-DA (10 mM) was applied to incubate H9c2 cardiomyocytes at 1°C for 37 h. As extracellular DCFH-DA was eliminated by washing, a flow cytometer was utilized for the detection of fluorescence intensity. The fluorescence excitation wavelength of 488 nm and emission wavelength of 525 nm were adopted. The percentage of control group was selected to present the level of intracellular ROS.

### Western blotting

The standard procedures were set as the criteria for Western blotting. Specifically, protein in equal amounts (40 μg) was aspirated from every sample and separated on gels for SDS-PAGE, followed by nitrocellulose membrane transfer. Subsequently, 5% skimmed milk was added to block the membrane. Afterward, the WesternBreeze color development immunoassay system was employed for blotting using anti-B-cell lymphoma-2 (Bcl-2) plus anti-Bcl-2-associated X protein (Bax) antibodies and GADPH. Lastly, Image Lab 4.0 software was employed for image capture and quantification.

### 4’,6-Diamidino-2-phenylindole (DAPI) staining

The following procedures were implemented for DAPI staining assay. Twice rinsing in cold PBS together with 20-min paraformaldehyde (4%) fixation were carried out for the harvested H9c2 cardiomyocytes, followed by PBS washing plus 37 min of DAPI (15 μM) incubation (1°C). After staining and rinsing, a fluorescent microscope was utilized for the photography of these cells.

### Annexin V/propidium iodide (PI) staining for apoptosis quantification

An Annexin V-FITC apoptosis assay kit was employed to detect apoptosis. Briefly, the collected cells (1×10^5^) were cleaned using ice-cold PBS twice, resuspended via 1× binding buffer supplemented with PE Annexin V plus 7-AAD (5 μL), and incubated under ambient temperature away from light for 15 min. Cells in the early stage of apoptosis were accurately identified by fluorophore-labeled membrane linker V (Annexin V), and those in the mid-late stages were stained using the fluorescent nucleic acid binding dye PI. Based on forward and side scatter gating, cell subsets were subjected to gating. The gated area of apoptosis rate was calculated using intermediate-large mononuclear cell populations. The apoptosis rate was the ratio of cell counts with negative PI and positive Annexin V on the membranes to the total cell count in the gated area.

### TUNEL assay

The TUNEL assay was performed to evaluate the apoptosis of H9c2 cells. After treatment, the cells were washed and fixed with 4% paraformaldehyde at room temperature. Following a 15-min incubation with proteinase K, the cells were placed in 3% H_2_O_2_ at room temperature for 15 min and then treated using a TUNEL detection kit. After incubation, the cells were counterstained with 1 μg/mL DAPI working solution for 10 min. The labeled H9c2 cells were observed using IX73 fluorescence microscope.

### Statistical analysis

Statistics analysis was accomplished by GraphPad Prism 7 software. Mean and standard deviation (SD) was determined as the expression format of all data which were compared using one-way analysis of variance among multiple groups. The statistically significant differences were indicated with p<0.05.

## RESULTS

### Culture and identification of H9c2 cardiomyocytes

Following 24 h of growth adhering to the wall, spontaneous throb of a single H9c2 cardiomyocyte was observed (Supplementary file Figure S1A). A daisy-like cell mass was observed after 72 h of growth (Supplementary file Figure S1B). Supplementary file Figure S1C shows the images observed under an inverted fluorescence microscope, in which red stood for specific staining of anti-cardiac troponin T and blue was DAPI-stained nuclei. H9c2 cardiomyocytes had a purity of over 98%, which can be used for subsequent experiments. Supplementary file Figure S1D displays the structure of cardiomyocyte filaments clearly observed by magnifying Supplementary file Figure S1C.

### Apelin-13 relieved nicotine-induced damage to H9c2 cardiomyocytes

**Figure 1:**
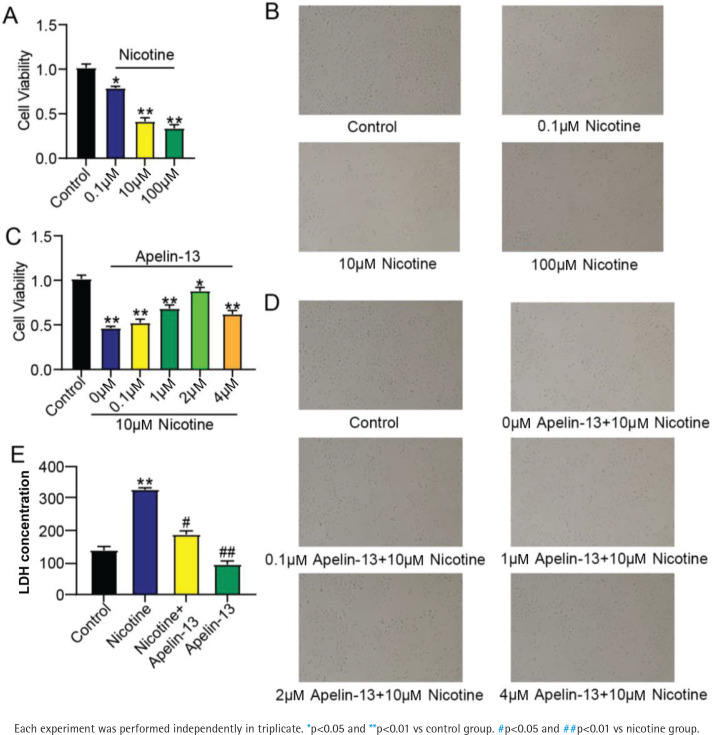
Effect of Apelin-13 on viability of H9c2 cardiomyocytes treated with nicotine: A and B) Toxicity of nicotine at various concentrations towards H9c2 cardiomyocytes; C and D) effects of Apelin-13 at various concentrations on H9c2 cardiomyocytes treated with 10 μM nicotine; E) LDH activity assay results

To verify the toxicity of nicotine on H9c2 cardiomyocytes, 0.1, 10 and 100 μM nicotine was used for 48 h of cell induction. The viability of H9c2 cardiomyocytes was significantly inhibited when the concentration of nicotine reached 10 μM, and the decrease in viability was not obvious when the concentration of nicotine rose (Figures 1A and 1B). Hence, 10 μM nicotine was selected for subsequent research. To determine the optimal concentration, H9c2 cardiomyocytes treated with 10 μM nicotine were cultured for 48 h by Apelin-13 at various concentrations. H9c2 cardiomyocytes were most viable when treated with 2 μM Apelin-13 (Figures 1C and 1D). Afterwards, the supernatant of H9c2 cardiomyocytes was collected and tested. LDH was increased by nicotine, but such an increase was evidently attenuated following treatment with Apelin-13 (Figure 1E). Taken together, Apelin-13 directly acted on H9c2 cardiomyocytes to relieve nicotine-induced damage.

### Effect of Apelin-13 on nicotine-induced anti-oxidant enzymes and lipid peroxidation in H9c2 cardiomyocytes

**Figure 2:**
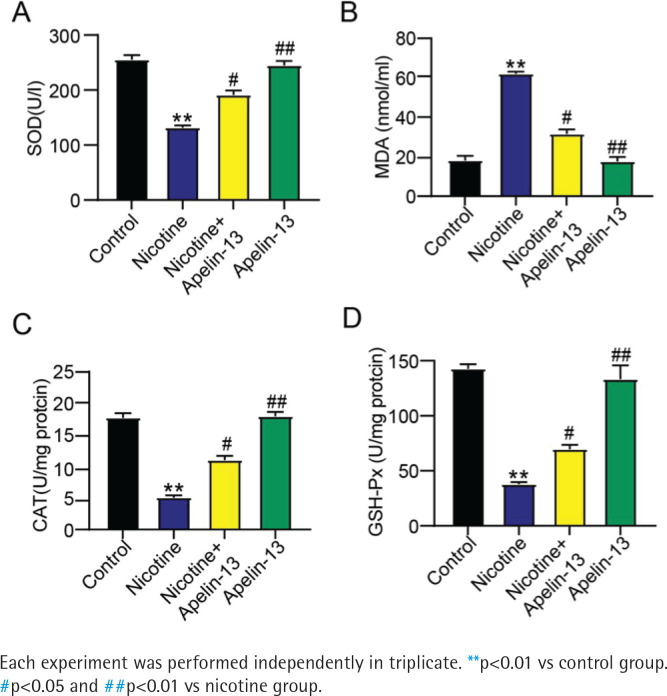
Effect of Apelin-13 on: A) SOD activity, B) MDA level, C) CAT activity and D) GSH-Px activity in H9c2 cardiomyocytes treated with nicotine

H9c2 cardiomyocyte lysate was examined to obtain the levels of lipid peroxidation products (e.g. MDA) besides anti-oxidant enzymes (including GSH-Px, SOD, and CAT). Compared to the control group, the nicotine group had significantly weakened activities of SOD, GSH-Px, and CAT, along with a significantly elevated MDA level (p<0.001). The MDA level declined, whereas CAT, GSH-Px, and SOD activities rose in the nicotine + Apelin-13 group in comparison to those in the nicotine group (p<0.05) (Figure 2). These outcomes verified a strong anti-oxidant capacity of Apelin-13 *in vitro*.

### Apelin-13 impeded ROS production in H9c2 cardiomyocytes treated with nicotine

**Figure 3:**
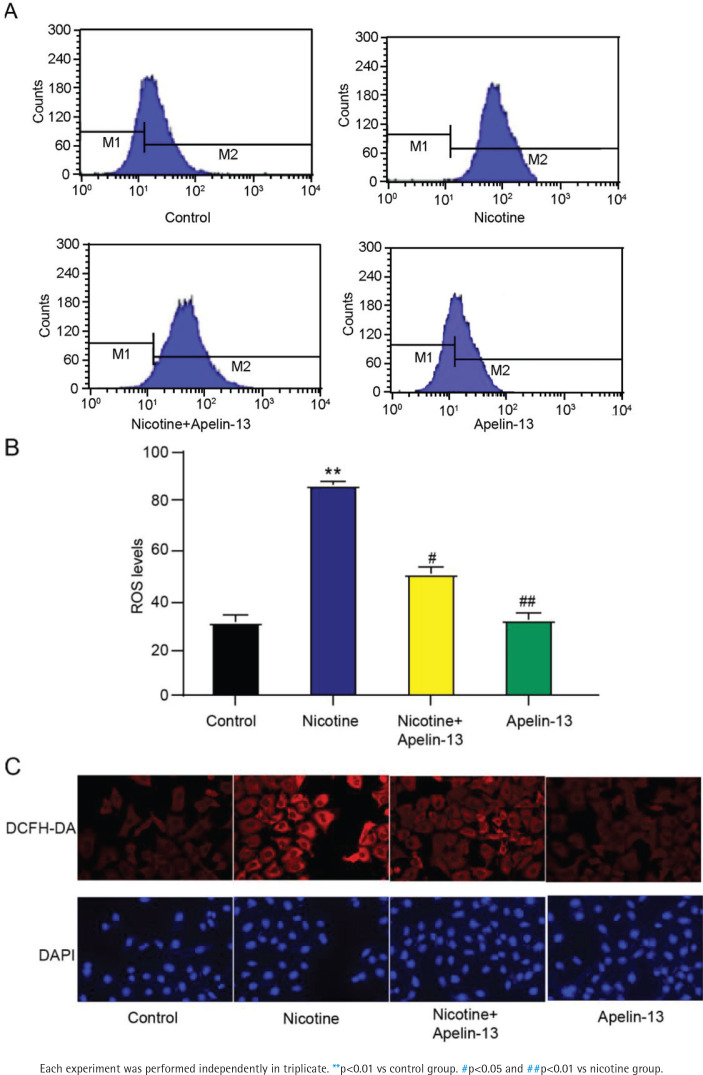
Effect of Apelin-13 on ROS production in H9c2 cardiomyocytes treated with nicotine: A) DCFH-DA assay results; B and C) ROS levels

The DCFH-DA assay was employed for the measurement of intracellular ROS level (Figure 3A). Compared with the control group, H9c2 cardiomyocytes treated with nicotine had a significantly increased ROS level (p<0.01). The nicotine + Apelin-13 group had a higher ROS level than that of the control group and a lower level than that of the nicotine group. No difference was found in ROS level between Apelin-13 and control groups (Figures 3B and 3C). Therefore, Apelin-13 may be an effective cardioprotective agent able to resist nicotine-induced oxidative stress.

### Apelin-13 suppressed inflammatory response in H9c2 cardiomyocytes treated with nicotine

**Figure 4:**
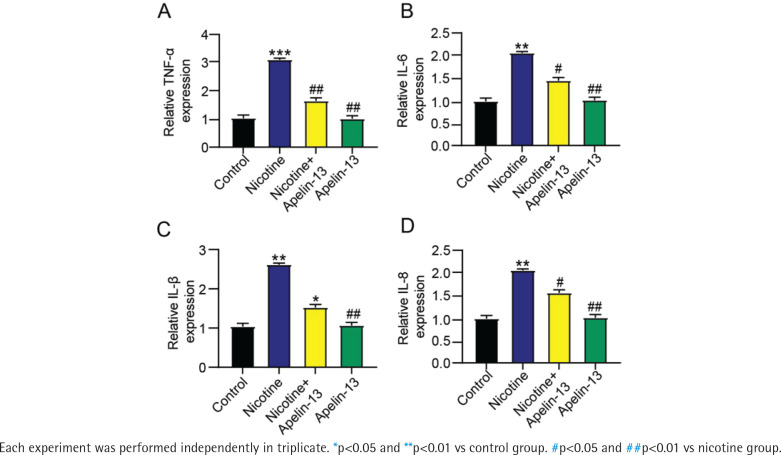
Effect of Apelin-13 on: A) TNF-α, B) IL-6, C) IL-β and D) IL-8 levels in H9c2 cardiomyocytes

In comparison to control and nicotine + Apelin-13 groups, the nicotine group had significantly higher activities of IL-8, TNF-α, IL-6, and IL-β, which were comparable between Apelin-13 and control groups (Figure 4).

### Morphology of apoptotic cardiomyocytes assessed by DAPI staining assay

**Figure 5:**
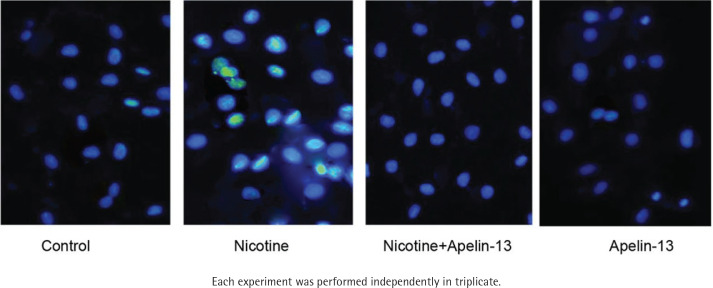
Effect of Apelin-13 on the morphology of H9c2 cardiomyocytes treated with nicotine

To study whether Apelin-13 protected H9c2 cardiomyocytes against apoptosis, the morphology of apoptotic cardiomyocytes was assessed using DAPI staining assay. The cardiomyocytes in the control group had light blue fluorescence, a round shape, complete membranes, as well as normal organelles. The cardiomyocytes treated with nicotine experienced shrinkage, solidification and lobulated nuclear cleavage, with bright blue fluorescence, but the above morphological changes were significantly alleviated in the cardiomyocytes cultured with Apelin-13 (Figure 5).

### Apelin-13 inhibited apoptosis of H9c2 cardiomyocytes treated with nicotine

The nicotine group had a significantly increased Bax protein level and a significantly decreased Bcl-2 level compared to those of the control group, suggesting that nicotine induced the apoptosis of cardiomyocytes. Compared with the nicotine group, H9c2 cardiomyocytes treated with Apelin-13 had a lowered Bax protein level and an elevated Bcl-2 level (Supplementary file Figure S2A). The results of flow cytometry also indicated that Apelin-13 protected cardiomyocytes against nicotine-induced apoptosis (Supplementary file Figure S2B). The results were further verified by the TUNEL assay (Supplementary file Figure S2C).

## DISCUSSION

The results of this study showed that Apelin-13 significantly suppressed ROS production, facilitated cell viability, attenuated the release of LDH and relieved nicotine-induced damage to H9c2 cardiomyocytes. Moreover, Apelin-13 prevented nicotine-induced damage to H9c2 cardiomyocytes.

SOD and CAT activities and GSH-Px level act as important lines of defense against free radicals^14^. As one of the end products of lipid peroxidation, MDA is able to induce severe cell damage by triggering the polymerization and cross-linking of membrane components, which is an indirect oxidative stress marker for cellular damage^15^. For this reason, SOD and CAT activities and GSH-Px and MDA levels are commonly applied to assess oxidative stress^16^. In the present study, Apelin-13 decreased MDA level, but enhanced the activities of SOD, CAT, and GSH-Px. These key anti-oxidant enzymes play crucial roles in ROS scavenging^17^. Overall, Apelin-13 prevented against the oxidative stress injury of H9c2 cardiomyocytes caused by nicotine, being consistent with the findings of a previous study^11^.

Under the conditions of normal physiology or pathology, apoptosis is modulated by autologous genes^18^. Recently, the apoptosis of cardiomyocytes has been considered a vital underlying mechanism leading to cardiac dysfunction. In this study, the morphology of cardiomyocytes changed, proving the apoptosis of H9c2 cardiomyocytes treated with nicotine. The apoptosis was characterized by cell contraction, DNA fragmentation, chromatin condensation and apoptotic bodies based on the results of DAPI staining assay^19^. Therefore, nicotine facilitated the apoptosis of H9c2 cardiomyocytes, which may be protected by Apelin-13. ROS-mediated oxidative stress can trigger the apoptosis of cardiomyocytes, which may be one of the main mechanisms of myocardial ischemic injuries^20^. Once produced, ROS can enhance the permeability of mitochondria, leading to the cytochrome C release. Such release is under the control of Bcl-2 family member proteins during oxidative stress in cardiomyocytes, and a higher Bcl-2 expression may suggest a higher cell survival rate, but only in the case of a low Bax expression, the cell survival rate rises^21^. Hence, the Bax/Bcl-2 ratio is an effective predictive factor for the apoptosis of cardiomyocytes^22^.

Nicotine induces many kinds of cells and animals to generate ROS, thus facilitating smoking-induced net oxidative stress^23^. For instance, Kim et al.^23^ found the function of nicotine in initiating proximal renal tubular cell (HK-2 cells) apoptosis. Additionally, Jaimes et al.^24^ reported that nicotine promoted ROS production in cultured thylakoid cells while stimulating fibronectin production besides cell proliferation. According to the study of Arany et al.^25^, the influence of TGF-β on proximal tubule cells in terms of α-SMA, fibronectin generation, and vimentin was strengthened by nicotine, implying the role of nicotine in facilitating epithelial-mesenchymal transition. Moreover, Lan et al.^26^ found that podocyte apoptosis under the stimulation of nicotine was mediated by ROS production and modulated by the p38, ERK1/2, and JNK pathways. Furthermore, Hu et al.^27^ reported that even short-term nicotine exposure elicited cardiac contractile and intracellular Ca^2+^ dysfunction.

Apelin-13 can relieve myocardial ischemic injuries in animal models^28^. Li et al.^29^ found that Apelin-13 treatment increased Akt/eNOS phosphorylation together with the expression of VEGF as a pro-survival signal and reduced myocardial apoptosis and infarct size at 24 h following ischemia, suggesting that Apelin-13 directly protected the ischemic heart. This direct protection against myocardial ischemic injuries by Apelin-13 may also be, at least in part, conducive to promoting the recovery of cardiac function in mice after myocardial infarction. Tao et al.^30^ reported that Apelin protected the heart by inhibiting endoplasmic reticulum stress in the case of diabetic cardiovascular complications. In addition, methylglyoxal-induced C/EBP-homologous protein and ATF4 protein expressions are inhibited by Apelin-13, demonstrating that Apelin-13 suppresses the induction of the endoplasmic reticulum-derived transcription factor to relieve methylglyoxal-triggered apoptosis^31^.

### Limitations

This study has some limitations. Firstly, the study utilized H9c2 cardiomyocyte cell line, which may not fully represent the complexity of cardiac tissue *in vivo* or in clinical practice. Secondly, this study used specific concentrations of nicotine (0.1, 10, and 100 μM) to induce damage. However, a broader range of concentrations can provide a more comprehensive understanding of the dose-dependent effects of nicotine on cardiomyocytes. Thirdly, further mechanistic studies are needed to elucidate the specific pathways through which Apelin-13 exerts its effects, such as signaling cascades or receptor interactions. Fourthly, the sample size is not large enough, and the long-term effects and potential toxicity of Apelin-13 are not evaluated. Further studies are ongoing in our group to confirm the findings.

## CONCLUSIONS

Apelin-13 significantly reduces nicotine-induced cytotoxicity, oxidative stress, and apoptosis in H9c2 cardiomyocytes. These results indicate that Apelin-13 exerts antioxidant and anti-apoptotic effects on this *in vitro* model. Further studies are needed to explore additional pathways involved in the protective effects of Apelin-13.

## Supplementary Material



## Data Availability

The data supporting this research are available from the authors on reasonable request.
