# Supplementary material for: Protective effects of Apelin-13 on nicotine-induced H9c2 cardiomyocyte apoptosis and oxidative stress
Source: Tob Induc Dis. 2025 Mar 18;23:10.18332/tid/201400. doi: 10.18332/tid/201400 (PMC11915093; doi:10.18332/tid/201400)
Supplement: Supplementary file 1 [file TID-23-33-s1.pdf]

Figure S1. Culture and identification of cardiomyocytes. A: Single H9c2 cardiomyocytes after 24 h of growth; B: daisy-like cell masses after 72 h of growth; C: inverted fluorescence microscope showed anti-cardiac troponin T stained in red and DAPI-stained nuclei in blue; D: cardiomyocyte filaments observed at high magnification.

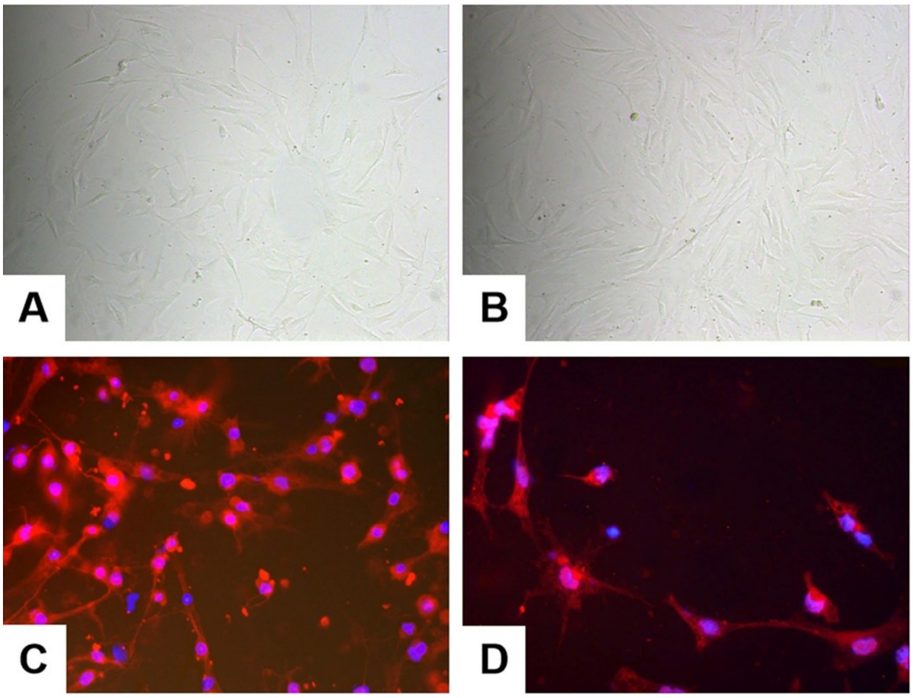

Figure S2. Effect of Apelin-13 on apoptosis of H9c2 cardiomyocytes treated with nicotine. A: Expressions of Bax and Bcl-2 measured through Western blotting; B: apoptosis rate detected by flow cytometry; C: TUNEL assay results. Each experiment was performed independently in triplicate. **P<0.01 *vs.* Control Group; $^{\#}$P<0.05 and $^{\#\#}$P<0.01 *vs.* Nicotine Group.

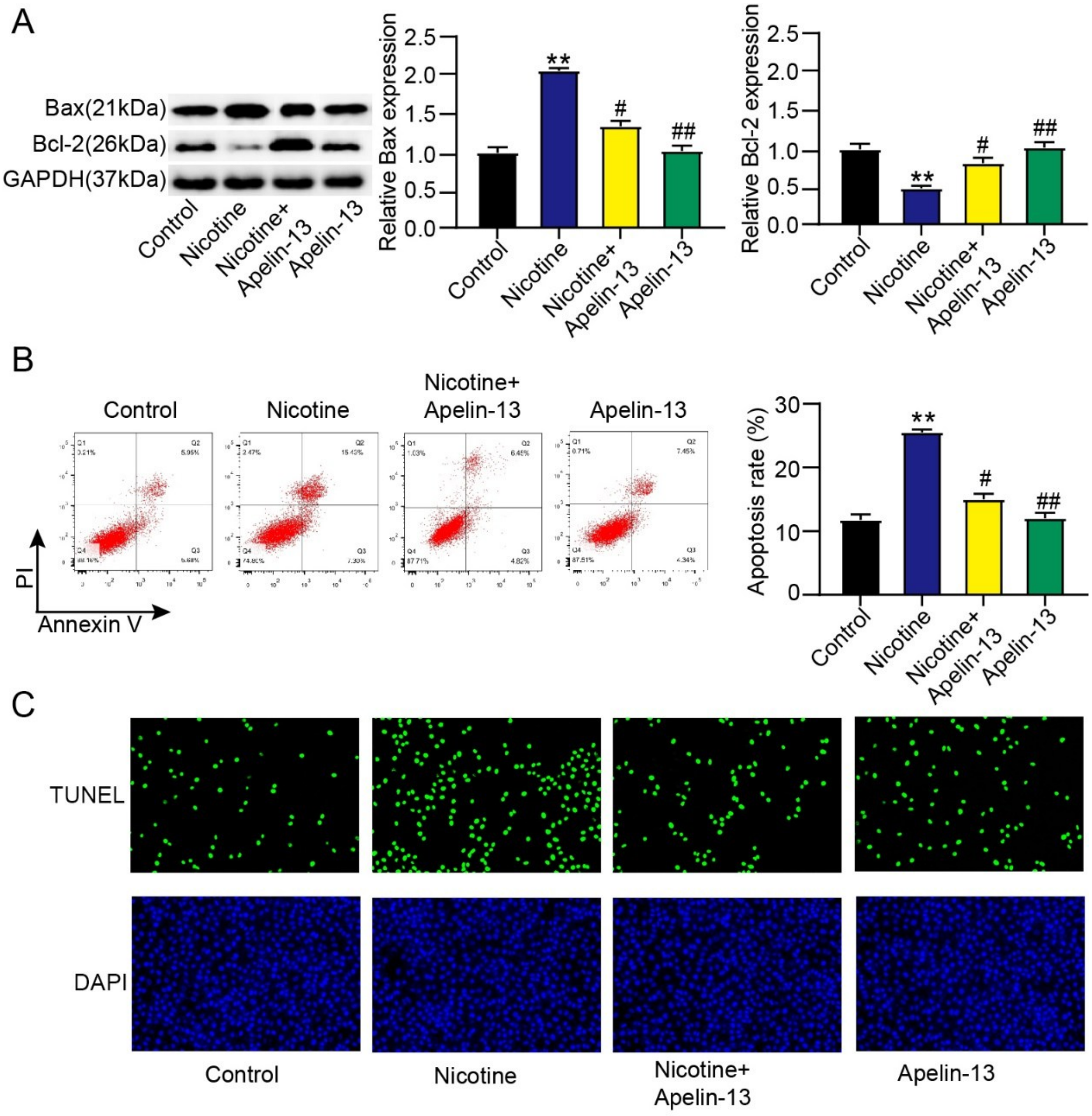

© 2025 Xu C. et al.
